# Current perceptions and use of paracetamol in dogs among veterinary surgeons working in the United Kingdom

**DOI:** 10.1002/vms3.1058

**Published:** 2023-02-16

**Authors:** Alba M. Bello, Charlotte Dye

**Affiliations:** ^1^ Department of Small Animal Medicine Pride Veterinary Centre IVC Evidensia Derby UK

**Keywords:** acetaminophen, analgesic, antipyretic, paracetamol, perception, safety, therapeutic

## Abstract

**Background:**

Paracetamol (acetaminophen) is recommended as a first‐line drug in the management of pain and pyrexia in humans due to its minor gastrointestinal, renal and vascular side effects.

**Objectives:**

To explore the perceptions and use of paracetamol in dogs by UK veterinarians.

**Methods:**

Veterinarians were invited to complete an online survey. Questions addressed career history, previous use, and personal perceptions of the use of paracetamol in dogs.

**Results:**

A total of 450 veterinarians were included in the survey; 96% (*n* = 431) of whom worked in small animal practice only. The majority reported a frequency of use of 1–3 times a week (*n* = 197), with oral dosing being the most common route of administration (*n* = 440). Only 8 had never prescribed paracetamol. Paracetamol was more commonly used to provide analgesia (*n* = 431) than for the treatment of pyrexia (*n* = 292) and was predominantly used as an alternative to NSAIDs (*n* = 408) or as part of multimodal analgesia (*n* = 406). Hepatotoxic (*n* = 311) and gastrointestinal (*n* = 120) side effects were a common concern, although the majority perceived a low risk of side effects and felt they were likely to be mild if they did occur (*n* = 279). Only 5% (*n* = 24) of respondents reported having experienced side effects, with gastrointestinal signs (*n* = 14) and hepatotoxicity (*n* = 6) being the most common.

**Conclusions:**

Paracetamol is widely used by veterinarians working in the UK. Most veterinarians have changed their perception on the use of paracetamol over the last decade and consider it to be associated with fewer side effects and with more effective analgesic and antipyretic effects than they had previously believed.

## Graphical abstract

Paracetamol is widely used by veterinarians working in the UK, with most clinicians having changed their perception of the use of this drug over the last decade. Paracetamol is considered to be associated with fewer side effects and with more effective analgesic and antipyretic effects than they had previously believed.

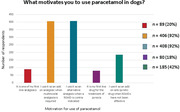

## 1 INTRODUCTION

Paracetamol (acetaminophen) is one of the most commonly used drugs in human medicine (Moore & Moore, 2016). It has a unique pharmacological profile that conveys potent analgesic and antipyretic effects with little or no anti‐inflammatory activity (Freo et al., 2021); the latter distinguishing it from non‐steroidal anti‐inflammatory drugs (NSAIDs) (Fadel et al., 2021).

In humans, it is recommended as a first‐line drug in the management of pain and pyrexia due to its minor gastrointestinal, renal and vascular side effects. It is also utilised in multimodal analgesia protocols due to its dose‐sparing effect when used in combination with other drugs such as non‐steroidal anti‐inflammatories and/or opioids (Freo et al., 2021; Hyllested et al., 2002; Westrich et al., 2019). While its antipyretic effects are mediated by cerebral COX inhibition (Ouellet & Percival, 2001), the mechanism underlying analgesia is poorly understood. Both peripheral and central nervous system sites of action have been postulated (Aronoff et al., 2006; Graham & Scott, 2005) and cholinergic, noradrenergic, opioid, and serotoninergic pathways have been suggested to be involved in its central, spinal and supraspinal actions (Freo et al., 2021).

There is a paucity of both controlled studies and case reports regarding the use of paracetamol in dogs, and further studies are required to elucidate its potential benefits and side effects in this species. As a result, current perceptions regarding the use of paracetamol in dogs are largely based on information translated from human studies, and from individual first‐hand experience of its use in both humans and dogs. Veterinary surgeons are therefore likely to have differing opinions about its use based on individual experience. Historically, the use of paracetamol in dogs was rarely advocated but, anecdotally, its use appears to have increased significantly over recent years. We therefore wanted to investigate whether paracetamol use is more common among vets who have graduated recently, and whether older vets are more likely to perceive negative connotations regarding its potential side effects.

This study surveyed the current perception and use of paracetamol in dogs by veterinary surgeons working in the United Kingdom.

## 2 MATERIAL AND METHODS

Electronic messages were sent to qualified veterinarians working with small animals and holding active membership of the Royal College of Veterinary Surgeons. They were invited to complete an online survey via the ‘Survey Monkey’ platform. Data collection and storage was done in accordance with the UK General Data Protection Regulations (GDPR).

The survey required informed consent and comprised 20 questions regarding career history (year and country of graduation, and current age), previous use of paracetamol in dogs (reasons and frequency of use, routes of administration and practice policy) and personal perceptions of the use of paracetamol in dogs (perceived efficacy, frequency and severity of side effects, and changes in perception of its use over the last decade). Responses were excluded if consent to share the data was not provided. Questions were considered invalid if concurrent incompatible answers were given.

In addition, printed surveys were distributed among veterinarians during veterinary meetings and conferences. These data were entered manually into the online platform.

Non‐parametric tests were used to analyse ordinal data and all other variables were assessed as categorical parameters. The Mann–Whitney *U* test was used for binary and ordinal data; the Kruskal–Wallis test was used for non‐directional ordinal data; the Jonckheere–Terpstra test was used for directional ordinal data (age categories, frequency of use and time of last use); and categorical data was analysed using the chi‐square test. *p* < 0.05 was deemed statistically significant.

## 3 RESULTS

A total of 454 questionnaires were completed of which 450 questionnaires fulfilled the criteria to be included in analysis.

### 3.1 Career history

Most of the veterinarians surveyed had qualified after 2010 (*n* = 232, 52%). For the remainder, there was a progressive reduction in the number of respondents with increasing time since graduation; 2000 to 2009 (*n* = 100, 22%), 1990 to 1999 (*n* = 74, 16%), 1980 to 1989 (*n* = 31, 7%), 1970 to 1979 (*n* = 9, 2%) and prior to 1969 (*n* = 3, 0.67%). One respondent did not provide this information. Three hundred and thirty‐six (75%) veterinarians had qualified in the United Kingdom and 113 abroad (25%). One veterinarian did not provide this information. Most of the surveyed veterinarians were 30 to 39 years old (*n* = 172, 38%). A total of 107 (24%) were 40–49 years old, 102 (23%) 20–29, 48 (11%) 50–59, 18 (4%) 60–69 and 3 (0.67%) were 70 years old or older.

Most of the surveyed veterinarians, 308 (68%) had no postgraduate qualifications. Sixty‐seven (15%) were certificate holders; 38 (8%) advanced practitioners and 18 (4%) were European and/or American specialists. Eleven (2%) respondents had an academic qualification such as a Master's Degree or a Doctor of Philosophy (PhD) and 4 (0.89%) had another type of qualification. Four (0.89%) respondents did not provide this information.

Four hundred and thirty‐one (96%) of the respondents worked in small animal practice only. Of these, 327 (73%) worked in first‐opinion clinical practice, 57 (13%) in practices with advanced practitioners and/or certificate holders and 47 (10%) in referral practices with European and/or American specialists. Fifteen (3%) respondents worked in mixed practice, 3 (0.67%) worked in academia and 1 (0.22%) veterinarian worked in another type of practice but did not provide this information.

### 3.2 Practice policy on the use of paracetamol

Most of the respondents (*n* = 314, 70%) worked in practices that did not have a specific policy on the use of paracetamol in dogs. Only 69 (15%) reported having a practice policy. Sixty‐five (14%) of the surveyed veterinarians were unsure and 2 (0.44%) respondents did not answer this question.

### 3.3 Use of paracetamol

**FIGURE 1:**
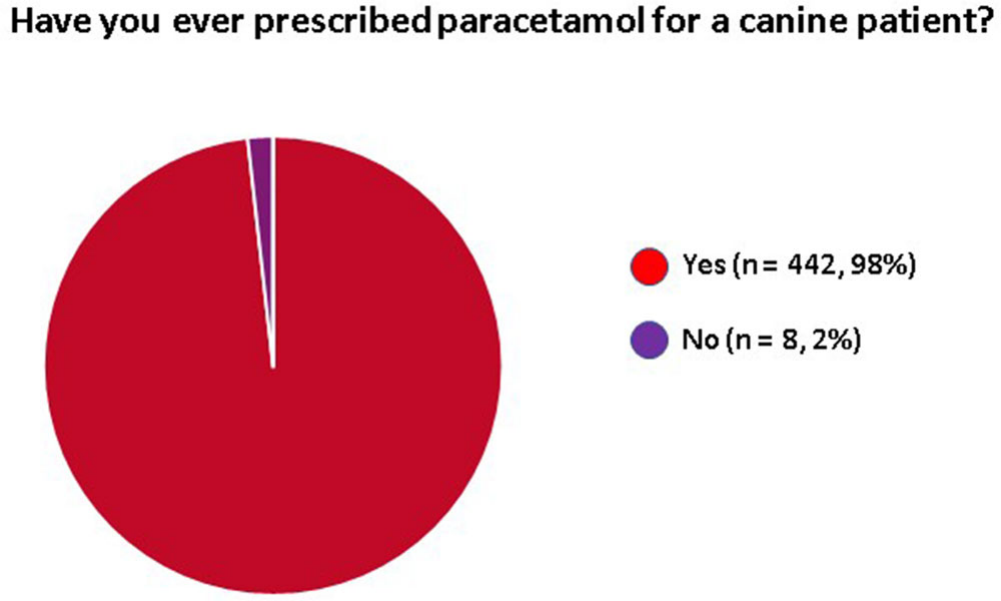
Pie chart illustration of the proportion of vets reporting to have prescribed paracetamol for dogs at some point during their career

Four hundred and forty‐two (98%) of the interviewed veterinarians had used paracetamol during their career (Figure 1). Among the 8 veterinarians that had never used paracetamol, reasons to have avoided it included: preference for other analgesic drugs with similar effects (*n* = 4), lack of awareness of its possible use in dogs (*n* = 2), perception of a high risk of side effects (*n* = 2), lack of familiarity with the drug (*n* = 1), lack of a licenced product (*n* = 1), lack of scientific evidence to support its use (*n* = 1) and its scarce use in their country of origin (*n* = 1).

Among the 442 veterinarians that had prescribed paracetamol, 400 (90%) had used the licenced oral formulation, containing paracetamol and codeine (Pardale‐V). Four hundred and fifteen (94%) respondents had used off‐licence formulations, containing paracetamol only. Oral preparations had been used by more respondents (*n* = 440, 99.55%) than intravenous preparations (*n* = 367, 83%).

**FIGURE 2:**
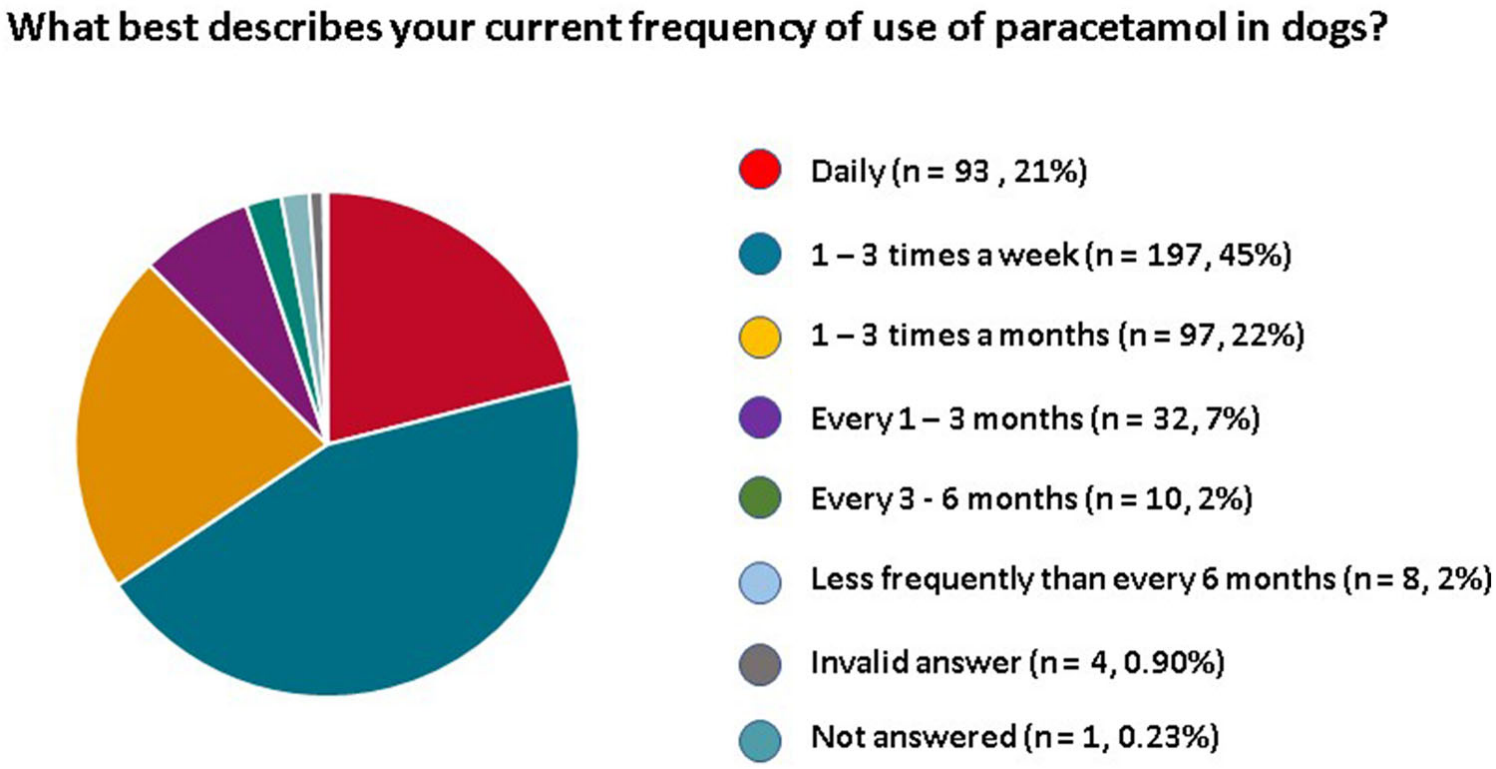
Pie chart illustration of the current frequency of use of paracetamol by veterinarians

Most respondents were using paracetamol frequently; daily (*n* = 93, 21%), 1–3 times a week (*n* = 197, 45%), 1–3 times a month (*n* = 97, 22%), every 1–3 months (*n* = 32, 7%), every 3–6 months (*n* = 10, 2%) and less frequently than every 6 months (*n* = 8, 2%). Four (0.90%) respondents gave an invalid answer and 1 (0.23%) did not complete this question (Figure 2).

Many of the veterinarians (*n* = 313, 71%) had prescribed paracetamol within the 7 days prior to completion of the survey. Ninety (20%) of the respondents had prescribed it within the previous month; 28 (6%) within the previous 3 months, and 3 (0.68%) within the previous 6 months. It had been more than 6 months since 7 (2%) of the surveyed veterinarians had prescribed paracetamol and 1 (0.23%) answer was invalid.

### 3.4 Perception on the efficacy and safety of paracetamol

**FIGURE 3:**
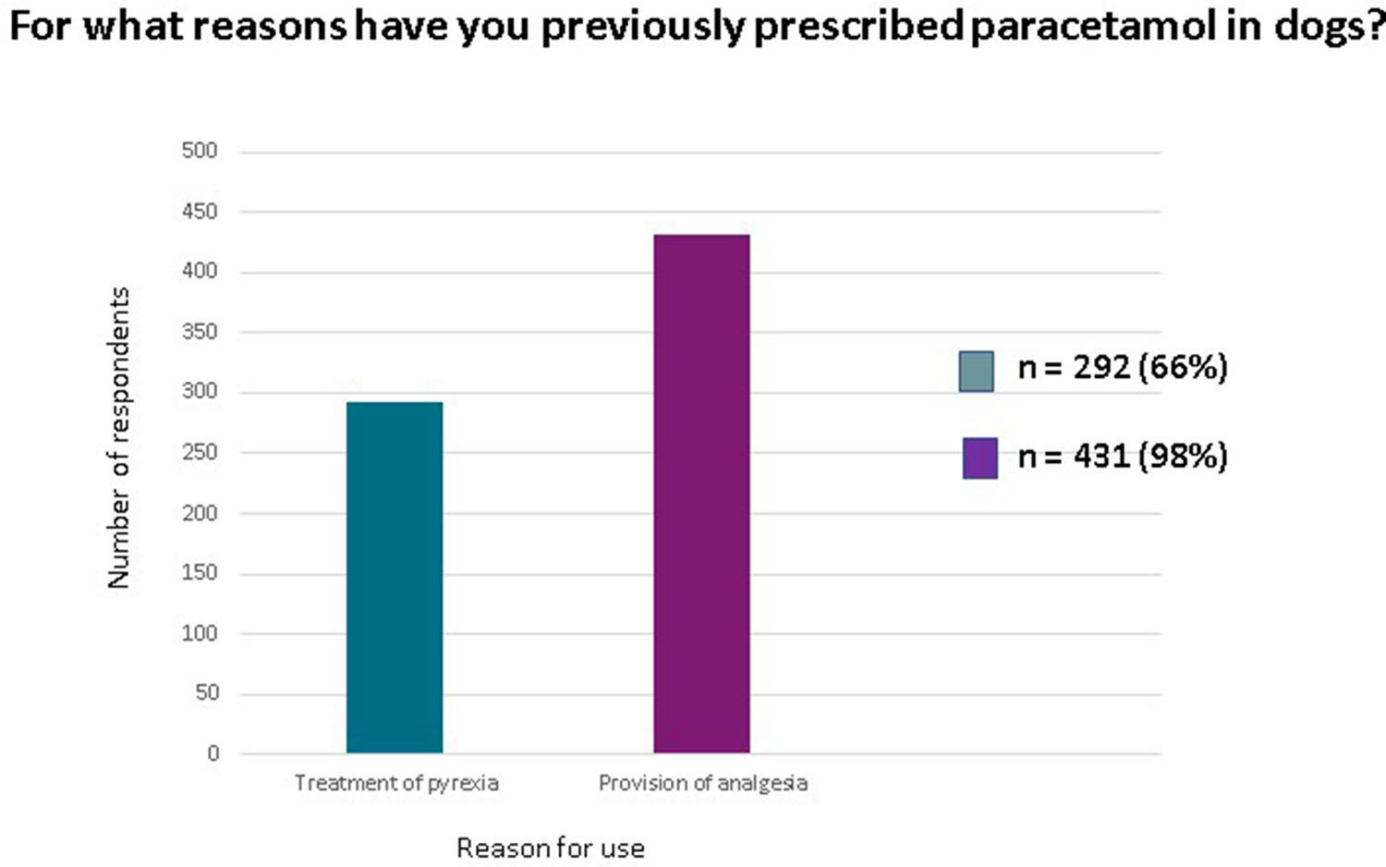
Histogram illustration of the number of veterinarians using paracetamol for its analgesic versus antipyretic properties

The most common reason to prescribe paracetamol was to provide analgesia (*n* = 431, 98%). In contrast, only 292 (66%) respondents used this drug for the treatment of pyrexia (Figure 3). Two respondents specifically reported its administration as part of their premedication protocol for caesareans, and one for the treatment of coughing. Two respondents did not answer this question.

**FIGURE 4:**
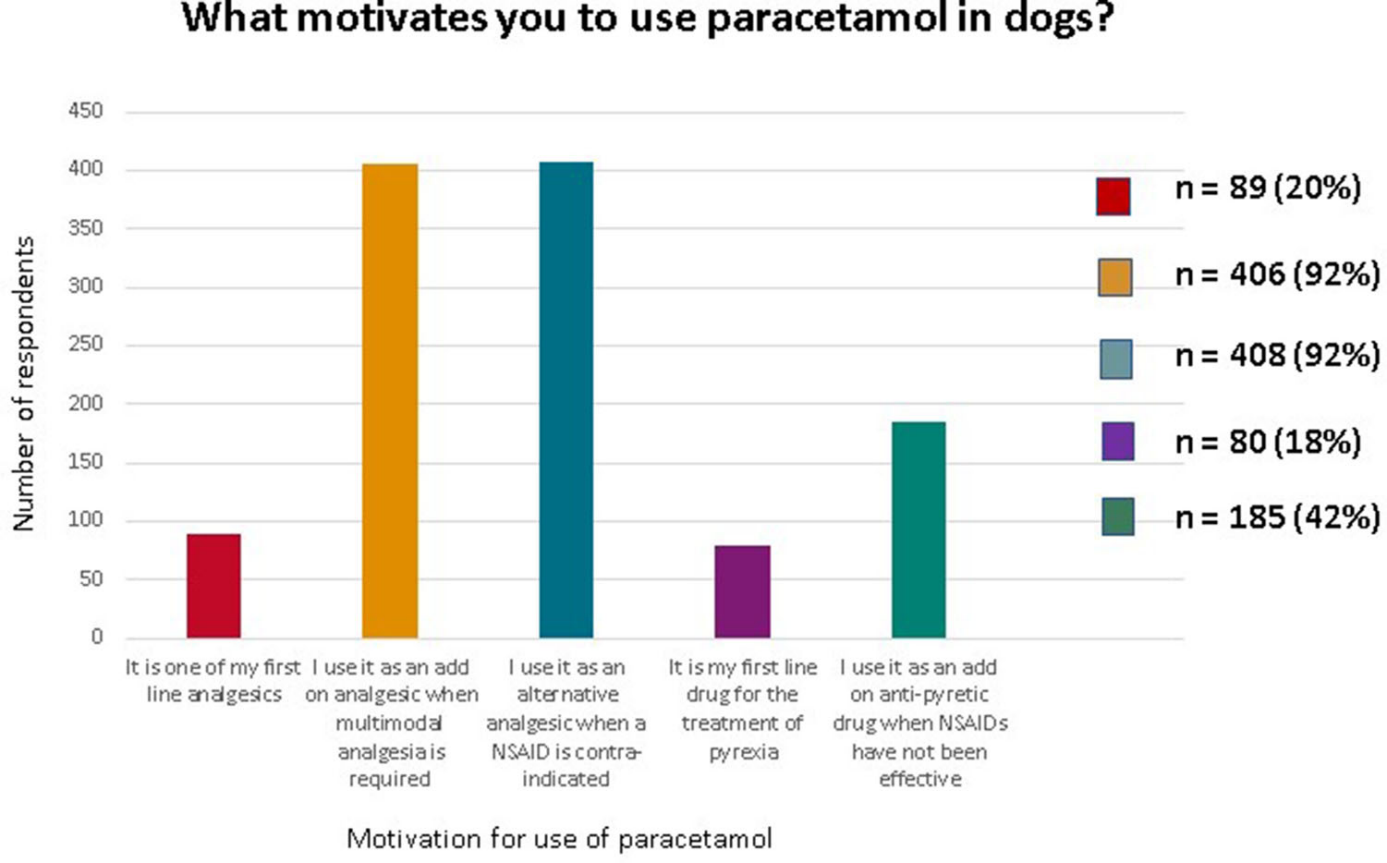
Histogram illustration of veterinarians’ motivations for prescribing paracetamol to dogs

Most respondents prescribed paracetamol as an alternative analgesic when non‐steroidal anti‐inflammatories were contra‐indicated (*n* = 408, 92%) and/or as part of multimodal analgesia (*n* = 406, 92%). One hundred and eighty‐five (42%) veterinarians reported using it as an add on antipyretic when non‐steroidal anti‐inflammatories had not been effective or were contraindicated. Eighty‐nine (20%) used it as one of their first line analgesic drugs and 80 (18%) used it as their first line drug for the treatment of pyrexia (Figure 4). Additional reasons to prescribe this drug included its low cost (*n* = 7, 2%) or because owners tended to already have it at home (*n* = 10, 2%), making it easily accessible when consulting remotely during the COVID‐19 lock‐down or when owners were calling out of hours. One (0.23%) respondent used paracetamol only if there were cost constraints and another (0.23%) used it only during remote consultations. One respondent gave an invalid answer.

**FIGURE 5:**
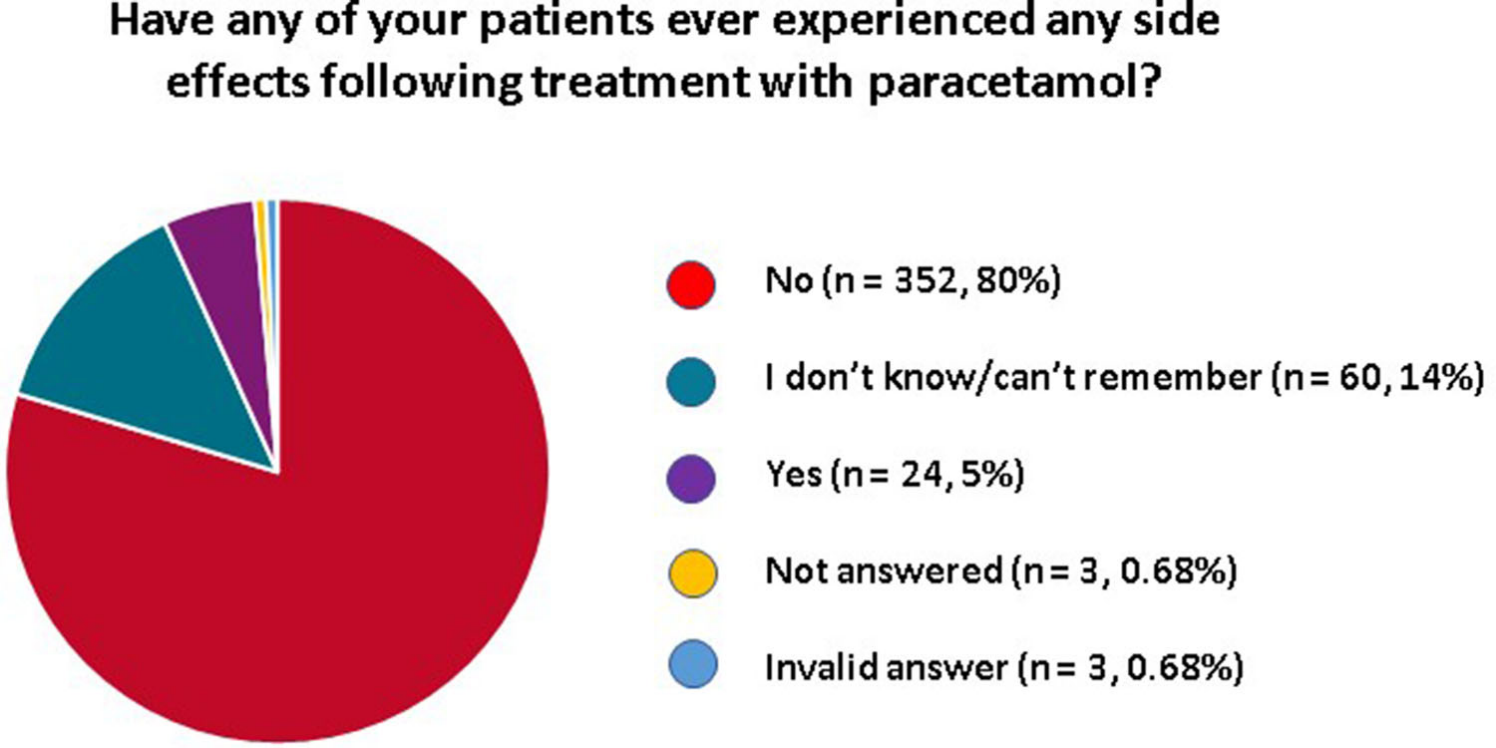
Pie chart illustration of the frequency of side effects experienced by veterinarians following paracetamol administration

Most respondents (*n* = 352, 80%) had never experienced any side effects following treatment with paracetamol. Only 24 (5%) had observed side effects and 60 (14%) were unsure or could not remember. Three respondents did not answer this question and 3 gave an invalid answer (Figure 5).

The most common side effects that had been experienced were gastrointestinal signs (*n* = 14, 58%) and hepatotoxicity (*n* = 6, 25%). Other reported side effects included sedation (*n* = 2, 8%), lethargy (*n* = 1, 4%), dysphoria (*n* = 1, 4%), hypersalivation (*n* = 1, 4%), constipation (*n* = 1, 4%), tachypnoea (*n* = 1, 4%) and polydipsia (*n* = 1, 4%).

**FIGURE 6:**
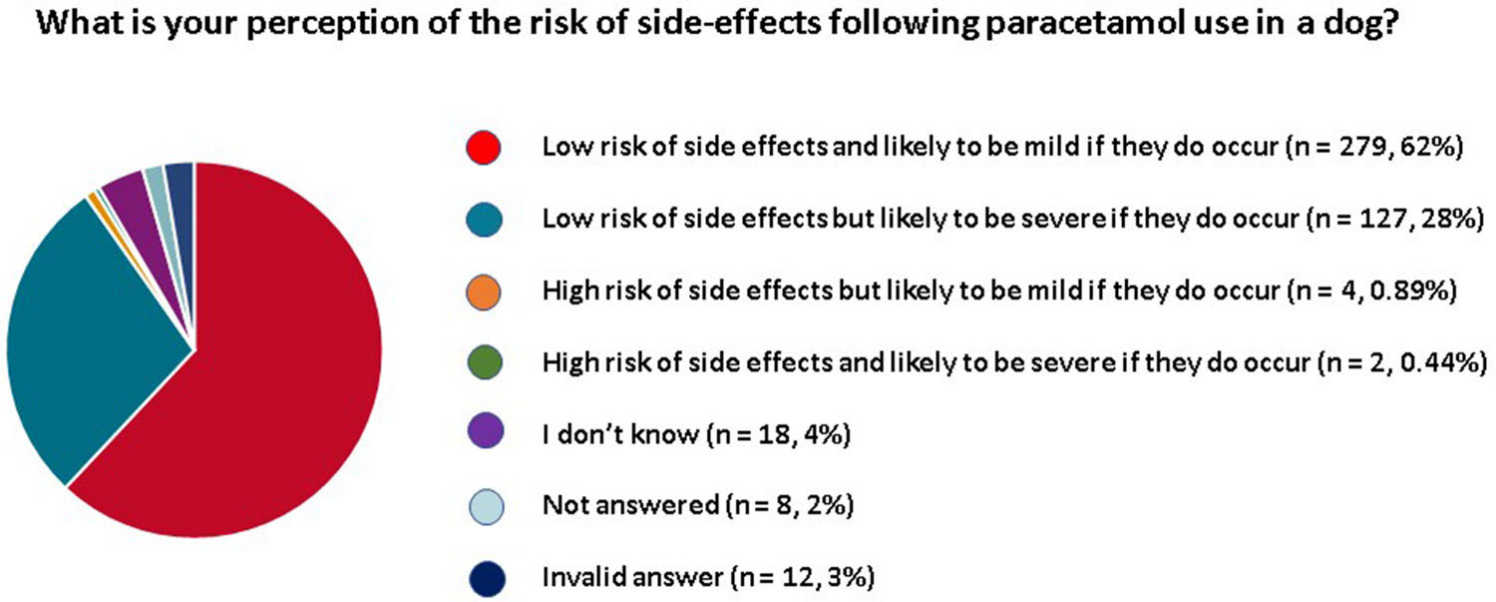
Pie chart illustration of veterinarians’ perceptions of the potential side effects associated with paracetamol administration in dogs

Among all 450 surveyed veterinarians, 279 (62%) perceived a low risk of side effects which were likely to be mild if they did occur. In contrast, 127 (28%) felt that paracetamol carried a low risk of side effects but that they were likely to be severe if they did occur. Only 6 (1%) respondents perceived a high risk of side effects, 4 of whom felt these were likely to be mild and 2 who believed they were likely to be severe if they did occur. Eighteen (4%) veterinarians did not have a strong opinion regarding the safety of paracetamol. Eight respondents did not answer this question and 12 answers were considered invalid (Figure 6). Both veterinarians that believed paracetamol had a high risk of side effects that were likely to be severe if they did occur had never prescribed paracetamol in their career. Among the other 6 veterinarians that had never prescribed paracetamol, 2 did not have a well‐established opinion regarding how risky it was to prescribe this drug, and the remaining 4 respondents did not answer this question.

Regarding the perception of risk, the predominant concerns were hepatotoxicity (*n* = 311, 69%) and gastrointestinal signs (*n* = 120, 27%). Other less commonly perceived side effects included: anaemia secondary to oxidative damage and methaemoglobinaemia (*n* = 18), renal disease (*n* = 18), bleeding tendencies (*n* = 10), abdominal pain (*n* = 6), allergic reactions (*n* = 6), lethargy (*n* = 4), hypersalivation (*n* = 4), respiratory signs (*n* = 4), nausea (*n* = 3), anorexia (*n* = 3), dry eye (*n* = 3), cardiac disease (*n* = 2), seizures (*n* = 2), constipation (*n* = 1), hypothermia (*n* = 1), polyuria‐polydipsia (*n* = 1), drug‐induced immune‐mediated disease (*n* = 1), weakness (*n* = 1), depression (*n* = 1), confusion (*n* = 1), tremors (*n* = 1), coma (*n* = 1), organ failure (*n* = 1) and death (*n* = 1). Sixty‐five respondents did not answer this question and 10 were unsure about the potential side effects of this drug. Six veterinarians felt there were no potential side effects of concern.

### 3.5 Likelihood of prescribing paracetamol

In univariate analysis, the age of veterinary surgeons was not associated with their frequency of paracetamol use (*p* = 0.111); however, as might be expected, age was related to postgraduate qualifications (*p* < 0.001) and qualification date (*p* < 0.001). In multivariate analysis accounting for postgraduate qualifications, qualification date, qualification in the United Kingdom or abroad, type of practice, perceived risk of side effects and whether or not side effects had been experienced previously, the age of veterinarians was associated with their frequency of use of paracetamol (*p* = 0.001) with a weak negative correlation (Pearson 0.178). In the multivariate analysis, postgraduate qualifications were associated with the frequency of use of paracetamol (*p* < 0.001) with a strong positive correlation (Pearson 0.764). Finally, the frequency of paracetamol use was higher in veterinarians who had qualified in the United Kingdom (*p* < 0.001). Interestingly, there was no difference in perception of risk regardless of age, whether or not side effects had been previously experienced, or the type of practice in which veterinarians were employed.

### 3.6 Changes in perception

**FIGURE 7:**
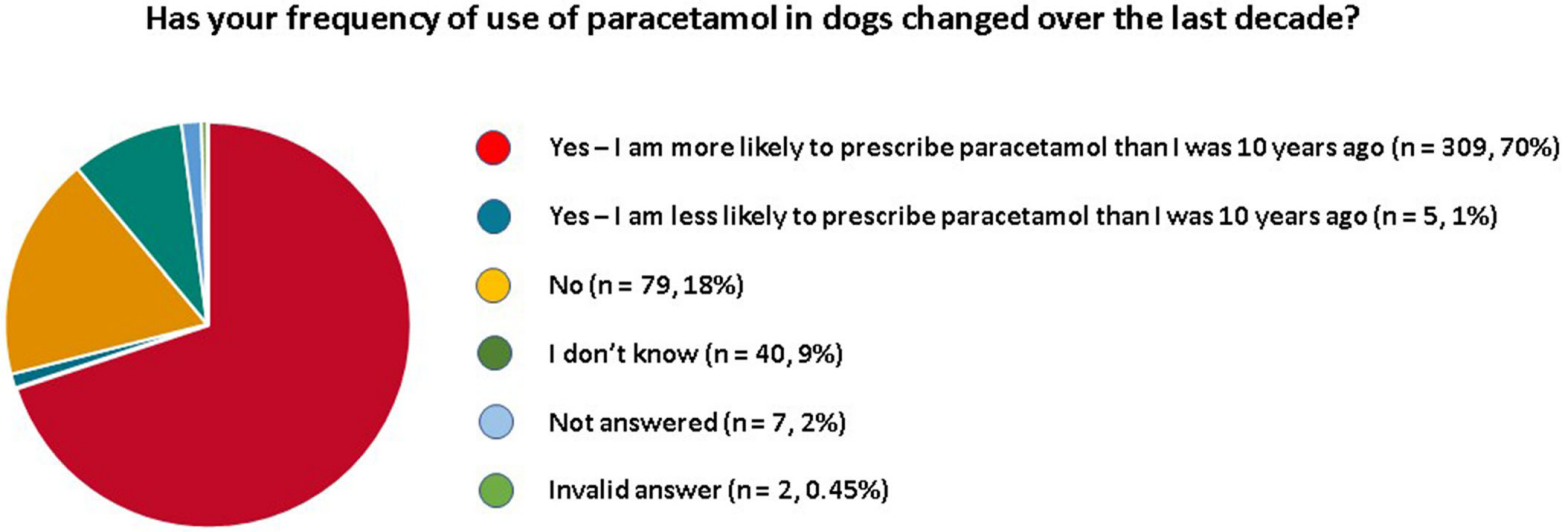
Pie chart illustration of the change in frequency of use of paracetamol in dogs by veterinarians over the last decade

Among the veterinarians that were using paracetamol in dogs, 314 (71%) had changed their frequency of use over the last decade. The vast majority, 309 (70%) of the respondents, were more likely to prescribe paracetamol now than 10 years ago. Only 5 (1%) were less likely to prescribe it. Seventy‐nine (18%) veterinarians had not changed their frequency of use over the last 10 years and 40 (9%) respondents were unsure. Seven veterinarians did not answer this question and 2 gave an invalid answer (Figure 7).

Among the 5 veterinarians reporting to have reduced their paracetamol usage over the last 10 years, only 1 had experienced side effects (reported as gastrointestinal signs). All 5 veterinarians considered the risk of side effects to be low, but two felt that side effects were likely to be severe if they did occur. Two of these five vets felt that their perception of paracetamol had not changed over the last decade. One felt that it was a less effective analgesic, one that it was a more effective analgesic, and the other that it was a more effective antipyretic than previously imagined.

**FIGURE 8:**
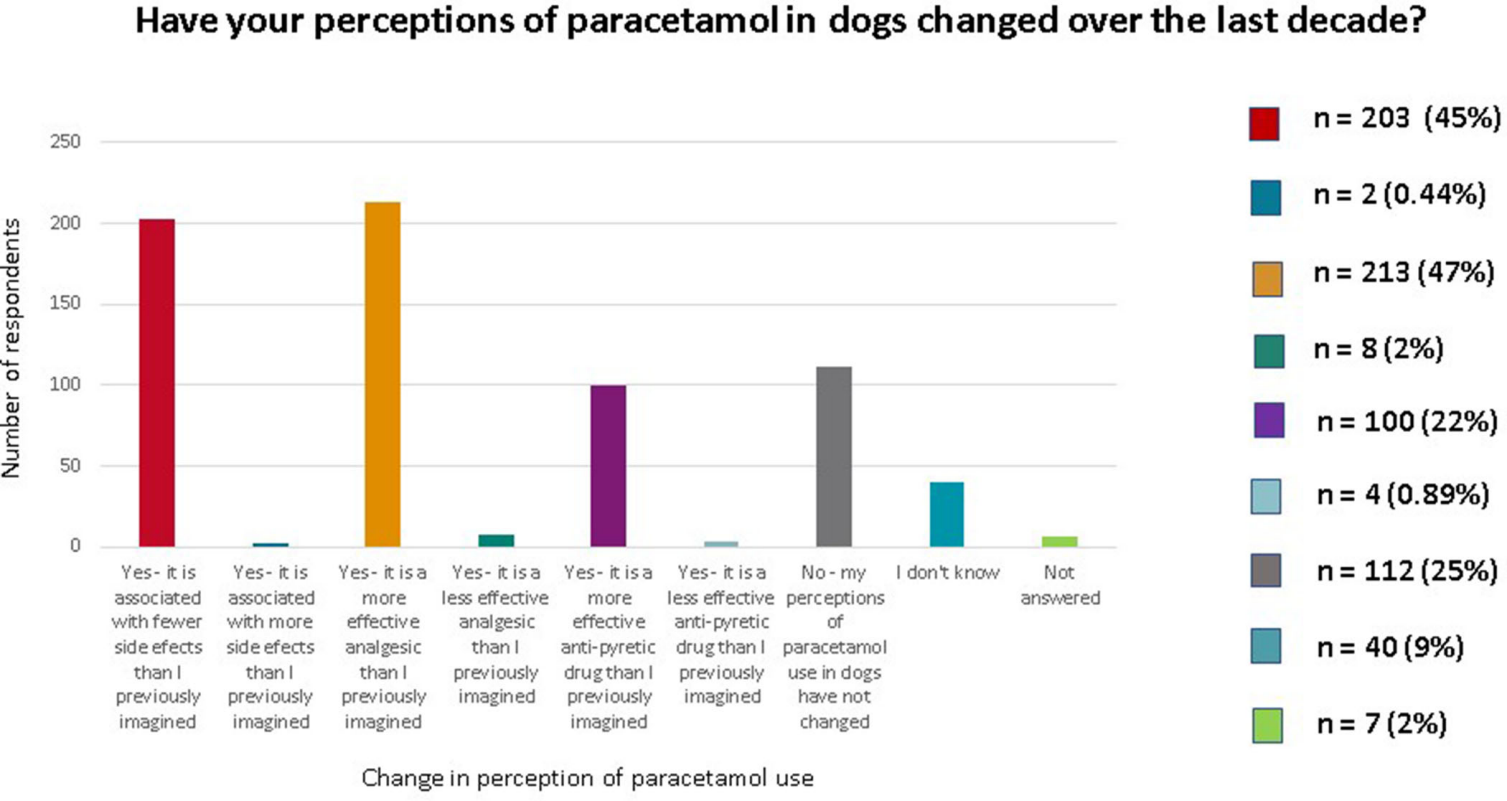
Histogram illustration of the change of veterinarians’ perceptions of paracetamol over the last decade

Among all surveyed veterinarians, most felt that paracetamol had a more effective analgesic effect (*n* = 213, 47%) and was associated with fewer side effects (*n* = 203, 45%) than they believed 10 years ago. Hundred (22%) vets felt it was a more effective antipyretic that they previously imagined. On the contrary, 8 (2%) respondents felt that paracetamol had less effective analgesic effects and 4 (0.89%) felt that it had less effective antipyretic effects than they had previously imagined. Only 2 (0.44%) veterinarians believed that it was associated with more side effects than they imagined 10 years ago; however, neither had experienced (*n* = 1) or remembered experiencing (*n* = 1) any side effects. 112 (25%) veterinarians had not changed their perception regarding the use of paracetamol in dogs. Forty (9%) were unsure if their perception had changed or had not worked in practice for enough time to have changed their opinion. Seven respondents did not answer this question (Figure 8).

## 4 DISCUSSION

In humans, paracetamol is a widely used drug and is considered to have good efficacy and few side effects (Freo et al., 2021). In dogs, there are no large studies evaluating its efficacy or safety, and its use is predominantly based on personal experience and/or the advice of colleagues. This study reveals that the use of paracetamol in dogs is nevertheless widespread in veterinarians in the United Kingdom. The results also suggest that paracetamol usage among veterinarians has increased over the last 10 years. A clear discrepancy with human medicine persists however, as demonstrated by the low number of veterinarians working in practices with well‐established policies on the use of this drug.

In human medicine, paracetamol is considered a first line drug for the management of chronic pain (Freo et al., 2021). In contrast, few veterinarians advocated the use of this drug as a first line analgesic, with most reserving it for part of a multimodal approach, or when other analgesic drugs were contraindicated. There was evidence that perceptions may be changing however, with almost half of the respondents believing that it was a more effective analgesic than they had considered it to be 10 years ago. Further prospective studies are required to evaluate the true pharmacological efficacy of this drug in the canine species.

Interestingly, one respondent reported the use of paracetamol for the treatment of coughing. Although a conclusion cannot be drawn, it is possible that this veterinarian was referring to the possible antitussive properties of the licensed form of paracetamol (Pardale‐V), which is combined with codeine.

Surprisingly, the generic off‐license form of paracetamol seems to be more commonly prescribed than the licensed form (Pardale‐V). Although this doesn't follow the United Kingdom prescribing cascade for veterinary medicines, it is possible that veterinarians are wanting to avoid the concurrent administration of codeine, which may be unnecessary in many situations. It is also possible that a shortage of the licensed product experienced over the last few years may have influenced the frequency of its prescription. Similarly, this study demonstrates how the veterinary profession has adapted and changed during the COVID‐19 pandemic, with respondents now recommending the use of this drug during remote consultations due to its wide availability. This may also have influenced the frequency of use of the unlicensed form.

In human medicine, toxicity resulting from paracetamol overdose is widely described (Major et al., 2016; Thusius et al., 2019); however, side effects are rare when it is used at the recommended therapeutic dose (Freo et al., 2021). Similarly, few veterinarians reported having experienced side effects following paracetamol administration. At the recommended dose of 10–15 mg/kg every 8 hours, potential adverse effects of paracetamol include renal, hepatic, gastrointestinal and haematological disorders. At higher doses (≥30 mg/kg), keraconjunctivitis sicca may also be induced (Plumb, 2018). In accordance with this, gastrointestinal and hepatic side effects were the most common side effects reported in this study. Respiratory distress, tachypnoea, cyanosis, anorexia, weight loss, lethargy, depression, hypothermia, weakness, oedema, facial swelling, hepatotoxicosis, abdominal discomfort and death are also mentioned in the literature following drug overdosage (Fadel et al., 2021; Nelson, 2019). Other potential side effects mentioned by the surveyed veterinarians included sedation, dysphoria, hypersalivation, constipation and polydipsia. These have not been previously cited in the veterinary literature but, if observed in the future, may need to be considered. It is likely, however, that some of these side effects may be related to the coadministration of codeine. Sedation and constipation are associated with codeine administration in dogs (Plumb, 2018) and these, as well as dysphoria, have also been reported in the human literature (British National Formulary, 2022).

In accordance with the author's hypothesis, the frequency of use of paracetamol was negatively associated with age in this study population. Although the correlation was weak, the discordant group sizes may have potentiated the risk of type 2 error.

Postgraduate qualifications had a strong positive correlation with the frequency of paracetamol use suggesting that vets with further qualifications may be more familiar with the pharmacological, therapeutic and safety profile of this drug. Interestingly, the frequency of paracetamol use was significantly higher among veterinarians who had qualified in the United Kingdom compared with vets who had qualified abroad. It is therefore possible that the availability, use and/or therapeutic knowledge regarding this drug among veterinarians may be more extensive in the United Kingdom. There is no data available comparing the frequency of use of paracetamol by humans in different countries; however, the UK Poisons Information Centre receives a high frequency of paracetamol related enquiries compared with other countries (Morthorst et al., 2017), suggesting that its use may be more widespread. Indeed, in the United Kingdom, paracetamol is a widely available over‐the‐counter drug that can be purchased from non‐pharmacy outlets. In contrast, paracetamol is only available from pharmacies in many European countries (Morthorst et al., 2017).

Limitations of this study include those associated with a survey‐based investigation such as misinterpretation of the questions and bias in replies from clinicians. Additionally, distribution of questionnaires via email may have preferentially targeted veterinary surgeons with wider internet availability, possibly resulting in a bias towards those with easier access to up‐to‐date drug information. The small number of veterinarians in some groups (for example the higher age and earlier graduation categories) may also have influenced the statistical analysis. In addition, no questions were asked regarding the specific situations in which paracetamol use was considered for the provision of analgesia. Veterinarians may therefore have had different perceptions depending on the perceived severity of pain or which body system was being targeted.

## 5 CONCLUSIONS

This study gives some insight into the current use and perception of paracetamol among first opinion and referral veterinarians in the United Kingdom. Paracetamol use appears to be widespread and is most often prescribed as part of a multimodal approach or when other drugs are contra‐indicated. The results suggest a change in perception of the drug over the last decade, with most veterinarians considering it to be associated with fewer side effects and with more effective analgesic and antipyretic properties than they had previously imagined.

## AUTHOR CONTRIBUTIONS

Investigation, data curation, formal analysis, visualisation, methodology, writing—original draft, preparation: Alba M Bello. Conceptualisation, investigation, formal analysis, visualisation, writing—review and editing, supervision: Charlotte Dye.

## FUNDING

IVC Evidensia for open access payment.

## CONFLICT OF INTEREST

The authors declare no conflict of interest.

### ETHICAL APPROVAL

This study was approved by the Research Ethics Committee of the University of Nottingham, United Kingdom. Data were collected and coded to ensure anonymity and confidentiality.

### PEER REVIEW

The peer review history for this article is available at https://publons.com/publon/10.1002/vms3.1058.

## Data Availability

The data that support the findings of this study are available on request from the corresponding author. The data are not publicly available due to privacy or ethical restrictions.
